# Risk factors for the onset and persistence of neck pain in undergraduate students: 1-year prospective cohort study

**DOI:** 10.1186/1471-2458-11-566

**Published:** 2011-07-15

**Authors:** Siriluck Kanchanomai, Prawit Janwantanakul, Praneet Pensri, Wiroj Jiamjarasrangsi

**Affiliations:** 1 Biomedical Sciences (Interdisciplinary Program) Graduate School, Chulalongkorn University, Bangkok, Thailand; 2 Department of Physical Therapy, Faculty of Allied Health Sciences, Chulalongkorn University, Bangkok, Thailand; 3 Department of Preventive and Social Medicine, Faculty of Medicine, Chulalongkorn University, Bangkok, Thailand

## Abstract

**Background:**

Although neck pain is common in young adulthood, studies on predictive factors for its onset and persistence are scarce. It is therefore important to identify possible risk factors among young adults so as to prevent the development of neck pain later in life.

**Methods:**

A prospective study was carried out in healthy undergraduate students. At baseline, a self-administered questionnaire and standardized physical examination were used to collect data on biopsychosocial factors. At 3, 6, 9, and 12 months thereafter, follow-up data were collected on the incidence of neck pain. Those who reported neck pain on ≥ 2 consecutive follow-ups were categorized as having persistent neck pain. Two regression models were built to analyze risk factors for the onset and persistence of neck pain.

**Results:**

Among the recruited sample of 684 students, 46% reported the onset of neck pain between baseline and 1-year follow-up, of whom 33% reported persistent neck pain. The onset of neck pain was associated with computer screen position not being level with the eyes and mouse position being self-rated as suitable. Factors that predicted persistence of neck pain were position of the keyboard being too high, use of computer for entertainment < 70% of total computer usage time, and students being in the second year of their studies.

**Conclusion:**

Neck pain is quite common among undergraduate students. This study found very few proposed risk factors that predicted onset and persistence of neck pain. The future health of undergraduate students deserves consideration. However, there is still much uncertainty about factors leading to neck pain and more research is needed on this topic.

## Background

Neck pain is common among adults, affecting 14-71% of adults at some point in their lives [1]. Its 1-year prevalence in adults ranges at 16-75% [1]. A substantial 19-37% proportion of neck pain patients will develop chronic neck pain [2,3]. Neck pain causes considerable personal discomfort due to pain, disability, and impaired quality of life, and may affect work. The economic consequences of treating disabling neck pain are significant [4-6]. Bernaard et al [7] recently postulated that the total yearly cost of neck and upper limb symptoms in the Netherlands due to decreased productivity, sick leave, chronic disability for work, and medical costs was 2.1 billion Euros.

Adolescents with neck pain are at high risk of having such symptoms in adulthood [8,9]. Life-long chronic neck pain may have its origins in childhood [10]. Thus to reduce the prevalence of neck pain in adults, knowledge regarding factors that can predict its onset and persistence in younger population is important [8].

Increasing evidence suggests a high prevalence of musculoskeletal symptoms in the neck and upper extremity among undergraduate students, ranging at 48-78% [8,11-13]. In a Swedish cohort of university students 15% developed neck or upper back pain during 1-year follow-up [14]. Neck pain is assumed of multi-factorial origin, indicating that individual, physical, and psychosocial factors can contribute to its onset and persistence [6,15]. In the general population McLean et al [16] systematically reviewed 14 prospective cohort studies and revealed that female sex, older age, high job demands, low social/work support, ex-smoker, and history of low back and neck disorders were linked to the onset of neck pain. In the working population, Côté et al [5] in their systematic literature review reported that risk factors associated with neck pain included age, previous musculoskeletal pain, high quantitative job demands, low social support at work, job insecurity, low physical capacity, poor computer workstation design and work posture, sedentary work position, repetitive work, and precision work. However, in undergraduate students only cross-sectional studies have been previously conducted on factors associated with neck and upper extremity pain [11,17,18]. It is not possible to establish causal relations between exposures and outcome in cross-sectional studies. Research to identify risk factors of neck pain requires longitudinal research design that permits tracking of study participants over time [19].

Computer use is very common among undergraduate students [18] and some epidemiological studies have been published with regard to its relation to onset of neck pain [20-22]. Undergraduate students involved in prolonged computer work and with high numbers of years of computer use more frequently reported upper extremity symptoms [11,12,23]. However, little is known about relations between computer use-related factors and onset and persistence of neck pain among undergraduate students.

There is also limited evidence of relations among clinical risk factors and neck pain. Most prior studies investigated the effects of biopsychosocial factors on neck pain in undergraduate students regardless of clinical factors such as muscle strength, endurance, and joint mobility [11,12,23]. Clinical factors may be a valuable diagnostic tool for early detection of musculoskeletal disorders [24-26]. Abnormal muscle strength, endurance, and joint mobility may lead to abnormal biomechanics of body movement, causing abnormal physical load to various tissues including muscles, ligaments, and bone. Thus individuals with abnormal muscle strength, endurance, and joint mobility may be susceptible to musculoskeletal injury [27].

The aims of this study were to examine the 1-year incidence and persistence of neck pain and to explore its biopsychosocial risk factors in undergraduate students.

## Methods

### Study Population and Procedures

A prospective cohort study with 1-year follow-up was conducted. At baseline, a self-administered questionnaire was sent to undergraduate students at Thammasat University aged 18-25 years. Subjects were excluded if they had reported neck pain in the previous 3 months, had any physician-diagnosed CNS/PNS neurological or musculoskeletal disorders, or history of upper extremity or spinal surgery. Those who were eligible for the study were invited to undergo a laboratory physical examination. The study was approved by Thammasat University Human Ethics Committee.

### Questionnaire

The self-administered questionnaire comprised three sections designed to gather data on individual, computer use-related, and psychosocial factors as well as neck pain.

Individual factors include sex, age (18-20 or 21-25 years), body mass index (< 18.5, ≥ 18.5- < 25, or ≥ 25 kg/m^2^), year of study (first to fifth year), chronic diseases (yes or no), field of study (art/humanities or science), and weekly frequency of exercise (regular, occasional, or no exercise).

Computer use-related factors include type of computer (desktop or notebook), years of computer use (< 5, 5-7, 8-9, or > 9 years) and average amount of daily computer use (< 3 or ≥ 3 hours/day). Respondents were asked whether during computer use their head, upper back, low back, and arms were supported, their feet flat on the floor, and their elbows, hips, knees, and ankles positioned at 90° flexion (yes or no). The questionnaire asked respondents, based on their own perceptions, to indicate the position of the computer screen (whether the top of the screen was positioned at a level horizontal with the eyes when they sat and looked straight ahead) and the appropriateness of the position of keyboard and mouse during computer use (suitable, too high, or too low). Respondents were asked about the percent time of computer use for study and entertainment as well as percentage duration using keyboard and mouse/touchpad (< 70% or ≥ 70%).

Psychosocial factors were assessed by Thai Mental Health Indicator Questionnaire (TMHI-15), a reliably validated instrument [28]. The questionnaire comprises 15 domains that assess general well-being, confidence in coping, kindness and altruism, self-esteem, and supporting factors. Each question is rated on four levels such as 0, completely disagree; 1, somewhat disagree; 2, somewhat agree; 3, completely agree. Respondents were asked whether the statements applied to them during the preceding 1 month. The total score of the test thus ranged from 0 to 45. The mental health score was scaled into three groups such as ≤27, worse than normal; 28-34, normal; 35-45, better than normal).

To assess neck pain during the previous 3 months, a picture of the body from the standardized Nordic questionnaire [29] and the question "Have you experienced any neck pain lasting > 24 hours during the past 3 months?" were included in the questionnaire.

### Physical examination

The physical examination was selected based on the hypothetical effect of prolonged computer use on body parts, which may lead to forward head posture, round shoulders and kyphotic upper thoracic spine [30,31]. These changes are likely to decrease neck mobility, neck muscle endurance, and nerve mobility as well as increase muscle tightness [32].

Each participant underwent a physical examination according to standardized protocol and the examiner was blinded to the questionnaire outcomes. Physical examinations included the following items and took a 45-minute single session to complete.

Neck range of motion assessment looked at active range of motion for neck flexion, extension, rotation, and lateral rotation using a Myrin goniometer [33]. In the starting position, each subject looked directly forward with the neck in a neutral position. The subject was then asked to move the head towards each direction as far as possible, and the degree of neck motion in each direction was recorded.

Pectoralis major muscle length was assessed according to guidelines described by Kendell and McCreary [34]. A subject's testing arm was positioned in approximately 135° shoulder abduction and lateral rotation with the elbow fully extended. The arm was dropped to table level with the low back remaining flat on a table. The examiners recorded the position of the arm relative to table level. To be regarded as normal, the extended arm should drop down to table level.

Neck extensor and flexor endurance were assessed according to the procedures described by Lee [35] and Harris et al [36], respectively. For the neck extensor muscles endurance test, a subject laid prone on a plinth with their head and neck over the edge of the plinth. The subject was instructed to hold the head steady in a position with the chin retracted and the cervical spine horizontal, monitored by a Myrin goniometer placed immediately above the tip of the right ear. The test was discontinued if the subject was not able to hold the position because of fatigue or pain, or if the subject lost > 5° of upper cervical spine retraction for > 5 seconds. The examiner recorded the muscle performance in seconds. For the neck flexor muscles endurance test, a subject lay on a plinth. The subject was instructed to lift the head until it was approximately 2.5 cm above the plinth while keeping the chin retracted to the chest. The test was discontinued if the subject's head touched the plinth for > 1 second. The examiner recorded the muscle performance in seconds.

Upper limb nerve tension was assessed according to the procedures described by Butler [37]. A subject lay supine on the plinth then the examiner depressed the shoulder girdle followed by abducting the shoulder 110°, flexing the elbow 90°, supinating the forearm, extending both wrist and finger, laterally rotating the shoulder, and extending the elbow. The examiner carefully extended the elbow to the point where the subject began to feel ache, pain, tingling, or discomfort. The range of elbow extension was measured by standard goniometer aligned along the mid-humeral shaft, medial epicondyle, and ulnar styloid.

Tertiles were used for categorizing the outcomes of neck range of motion, neck flexor endurance, and nerve tension tests. The outcome from the neck extensor endurance test had very low variance among the study sample; thus it was scaled into two groups based on mean score (522 seconds). The outcome of the pectoralis major muscle length assessment was classified into two groups: normal or restricted length.

Repeatability of data from the questionnaire and physical examination was assessed in 20 undergraduate students. Each subject was tested on two occasions separated by an interim of 7 days between measurements for the questionnaire and 1 day for the physical examination.

### Follow-up

Subjects were followed every 3 months for 12 months by telephone. The yes/no question "Have you experienced any neck pain lasting > 24 hours during the past 3 months?" was asked at each follow-up. If they answered "Yes", follow-up questions about the cause of neck pain were asked. Subjects who reported an accident preceding the neck pain episode or physician diagnosis of congenital anomaly of the spine, rheumatoid arthritis, infection of the spine and discs, ankylosing spondylitis, cervical spondylosis, malignant tumor, systemic lupus erythematosus, or osteoporosis were excluded from the study. Those who reported neck pain for ≥ 2 consecutive follow-ups were categorized as having persistent neck pain.

### Statistical analyses

For the reliability study of questionnaire outcomes, the intraclass correlation coefficient (ICC [1,1]) was calculated for continuous data and Spearman's rho (ρ) for nominal and ordinal data. ICC (1,1) was calculated for physical examination outcomes.

Subject characteristics were described by means or proportions. Percent missing data in individual, computer use-related, and psychosocial factor categories was 0.1%, 1.3%, and 0.2%, respectively. To retain the statistical power of the database, missing data were handled by "hot-deck imputation" procedure. A respondent was selected at random from the total sample of the study and the value for that person was assigned to the case in which information was missing. This procedure was conducted repeatedly for each missing value until the dataset was complete [38].

Two regression models were built to analyze risk factors for onset and persistence of neck pain. Initially, univariate analysis was carried out first to determine significant differences in the onset and persistence of neck pain with various biopsychosocial characteristics. Separate multivariable logistic regression models were then performed to assess associations between onset and persistence of neck pain and biopsychosocial factors. Backward selection procedures were used in the statistical modeling. Any factors with p-value ≤0.2 in the univariate analysis were eligible for addition into the modeling procedures. Odds ratios (OR) associated with particular factors were adjusted for the effect of all other factors in the model. Adjusted ORs and 95%CI for the final models are presented. Statistical significance was set at the 5% level. All statistical analyses were performed using SPSS statistical software, version 17.0 (SPSS Inc, Chicago, IL, USA).

## Results

The reliability results demonstrated moderate (0.60) to excellent (1.00) repeatability for questionnaire outcomes and moderate (0.71) to excellent (0.86) for physical examination outcomes.

Among a total of 3545 students who received the questionnaire 2614 responded (response rate, 73.7%). Of these, 103 were excluded because they did not meet inclusion criteria, giving an eligible population of 2511. In total, 684 students agreed to participate in the physical examination. Five hundred twenty-four (77%) students were followed for 1 year and 160 (23%) students could not be contacted during the follow-up period. Table 1 presents the demographic characteristics of the study population. A total of 239 (46%) students reported new onset of neck pain during the follow-up, of whom 79 (33%) reported persistent neck pain.

**Table 1:** Characteristics of undergraduate students (n = 524)

Characteristic	N	%	Mean	SD
***Sex***				
-Male	138	26.3		
-Female	386	73.7		
***Age (years)***			19.4	1.1
***Year of study***				
-Year 1	183	34.9		
-Year 2	247	47.1		
-Year 3	91	17.4		
-Year 4	0	0		
-Year 5	3	0.6		
***Field of study***				
- Arts/humanities	175	33.4		
- Science/health science	349	66.6		
***Hours of daily computer use***			2.9	1.8

### Risk factors for neck pain

According to univariate analyses factors showing p-value < 0.2 were neck extension range of motion, right upper limb tension outcome, positions of elbows, knees, and ankles during computer use, upper back and elbow support, positions of computer screen, keyboard, and mouse and percent duration of mouse use. Thus these factors were selected for further analysis. Multivariable logistic regression analyses revealed that positions of computer screen and mouse were associated with new-onset neck pain (Table 2).

**Table 2:** Incidence of neck pain and adjusted odds ratio (ORadj) with 95% confidence intervals (95%CI) with respect to factors in the final modeling (n = 524)

Factors	N	Incidence n (%)	OR_adj_	95%CI	*P*
*Elbows positioned at 90° angle *					
- Yes	132	49 (37.1)	1.00		
- No	392	190 (48.5)	1.52	0.99-2.31	0.052
*Computer screen is positioned at a level horizontal with the eyes*					
- Yes	221	85 (38.5)	1.00		
- No	303	154 (50.8)	1.64	1.13-2.36	0.008*
*Mouse height*					
- Suitable	382	172 (45)	1.00		
- Too high	93	50 (53.8)	1.30	0.82-2.10	0.272
- Too low	49	17 (34.7)	0.52	0.28-0.99	0.045*
*Percent duration of mouse use during desktop*					
- < 70	427	201 (47.1)	1.00		
- ≥ 70	97	38 (39.2)	0.66	0.42-1.04	0.075

Significance and OR_adj _with 95%CI from the multivariate analysis

*P ≤ 0.05

Students reporting that computer screen position was not level with the eyes were at greater risk of developing neck pain than those reporting that computer screen position was level with the eyes (adjusted OR, 1.64; 95%CI, 1.13-2.36).

Students reporting that the mouse position was too low were at lower risk of developing neck pain than those reporting mouse position suitable (adjusted OR, 0.52; 95%CI, 0.28-0.99).

### Risk factors for the persistence of neck pain

According to univariate analyses factors showing p-value < 0.2 were neck flexor endurance, year of study, percent time of computer use for entertainment, positions of elbows and ankles during computer use, positions of computer screen, keyboard, and mouse. Thus these factors were selected for further analysis. Multivariable logistic regression analyses revealed that year of study, percent time of computer use for entertainment, and positions of keyboard were associated with persistent neck pain (Table 3).

**Table 3:** Rate of persistent neck pain and adjusted odds ratio (ORadj) with 95% confidence intervals (95%CI) with respect to factors in the final modeling (n = 524)

Factors	N	Persistent rate n (%)	OR_adj_	95%CI	*P*
*Year of study*					
- 1^st ^year	183	21 (11.5)	1.00		
- 2^nd ^year	247	47 (19.0)	1.90	1.08-3.35	0.027*
- 3^rd ^year	91	10 (11.0)	0.96	0.42-2.15	0.912
- 5^th ^year	3	1 (33.3)	7.09	0.57-87.70	0.127
*Percent time of computer use for entertainment*					
- < 70	426	70 (16.4)	1.00		
- ≥ 70	98	7 (9.2)	0.44	0.21-0.95	0.036*
*Elbows positioned at 90° angle*					
- Yes	129	13 (10.1)	1.00		
- No	395	66 (16.7)	1.76	0.92-3.35	0.087
*Keyboard height*					
- Suitable	370	52 (14.1)	1.00		
- Too high	85	22 (25.9)	2.18	1.21-3.91	0.009*
- Too low	69	5 (7.2)	0.46	0.17-1.20	0.113

Significance and OR_adj _with 95%CI from multivariate analysis

*P< 0.05.

Second year students were at significantly higher risk of experiencing persistent neck pain than first year students (adjusted OR, 1.90; 95%CI, 1.08-3.35).

Students who reported using a computer for entertainment ≥ 70% were at lower risk of experiencing persistent neck pain compared with whose use was < 70% (adjusted OR, 0.44; 95%CI, 0.21-0.95).

Students reporting that keyboard position was too high were at greater risk of experiencing persistent neck pain than those reporting it was suitable (adjusted OR, 2.18; 95%CI, 1.21-3.91).

## Discussion

The principle aim of the present study was to determine the 1-year incidence and persistence of neck pain among undergraduate students. The 1-year incidence of neck pain among undergraduate students in this study was high, at 46% among whom 33% reported persistent neck pain. Grimby-Ekman et al [14], on the other hand, reported that the annual incidence of neck or upper back pain in Swedish undergraduate students was 15%. The discrepancy between our and previous studies may be due to difference in frequency of data collection during follow-up and the definition of symptomatic case. Grimby-Ekman et al [14] followed their subjects on a yearly basis, whereas in this study subjects were followed every 3 months for ≤1 year. Data collection every 3 months would reduce the influence of recall bias. Also, in the previous study a symptomatic case was defined as an individual who experienced pain for > 7 days whereas in this study it was > 24 hours. Consequently, it is likely that a far greater number of subjects were identified as symptomatic cases in this study.

Neck pain is regarded as a chronic episodic condition characterized by persistent, recurrent, or fluctuating pain and disability [3]. Earlier studies showed that persistent musculoskeletal symptoms were common among young population [8,14,39]. Slightly more than one quarter of our study sample who reported new onset of neck pain experienced persistent neck pain. In this study, persistent neck pain was defined as that reported for ≥ 2 consecutive follow-ups. There is a lack of consensus over the operational definition of persistent musculoskeletal pain. Stanton et al [40] defined recurrence as persistence of pain reported both at baseline and follow-up assessment with no recovery, whereas Hill et al [41] proposed that persistent pain could reflect chronic, recurrent, or continuous pain. In the present study, we did not ask subjects whether they had experienced any recovery period of pain since they previously reported symptoms. Thus it is not possible to differentiate those who had recurrent pain from those who had continuous pain. However, within this limitation, our findings suggest that students with neck pain may become symptomatic adults, highlighting an urgent need for stakeholders to pay more attention to the problem of neck pain in the young population to reduce the impact of neck pain later in life.

A secondary aim of this study was to identify risk factors for onset and persistence of neck pain. Computer use-related factors contributed significantly to these. However, risk factors for onset differed from those for persistence of neck pain, as was found in previous studies [8,42]. Persistent pain can have broad and profound effects on well-being with significant impairment of physical and psychological health [43]. Thus information about risk factors for persistent neck pain is of considerable importance.

Onset of neck pain was predicted by the computer screen position not being level with the eyes. High computer screen height results in the neck being more erect [44]; simultaneous increase in muscle activity of the neck extensor and sternocleidomastoid muscle was reported in this posture, and therefore prolonged computer use in this posture may be harmful [45]. The effect of low computer screen is inconclusive. Studies showed that low computer screen height increases neck flexion, neck extensor activities and compressive loading of neck ligaments, joint capsules, and other structures of the cervical spine, thus possibly increasing musculoskeletal strain in the upper body [46-49]. However, Fostervold et al [50] showed that working with low monitor screen height improved oculomotor status with significant reductions in musculoskeletal symptoms in the upper body. In the present study respondents were asked only whether the top of the computer screen was positioned at a level horizontal with the eyes when they sat and looked straight ahead. Further study is required to evaluate the relation between high/low computer screen height and neck pain in undergraduate students.

A mouse position self-rated as too low decreased the risk of onset of neck pain. This finding is contrary to the common concept of "good computer posture" often described as a position in which the upper arm is perpendicular to the floor, the elbow at a right angle, and forearm parallel to the floor [51]. However, Marcus et al [52] showed that elbow angles between 137° and 148° while using the mouse were correlated to lower risk of developing neck and shoulder pain in newly hired computer workers. Although the exact elbow angle while using a mouse was not measured in the present study, it is plausible that mouse position self-reported as too low may correlate with elbow angles > 90°. This hypothesis warrants further investigation.

Factors that predicted persistence of neck pain were students being in the second year of their studies, use of computer for entertainment < 70% of total computer usage time, and position of the keyboard being too high. The risk of persistent neck pain was 1.9-fold higher for second year students in comparison with first year students. Ndetan et al [53] found that chiropractic students are predominantly exposed to injury risk factors during the first, third, and sixth academic trimesters. However, the finding may not necessarily reflect other student groups. To our knowledge, this study is the first to demonstrate that year of undergraduate study significantly correlated with neck pain. This information may be of importance for developing viable prevention strategies of neck pain in young population. Further study is needed to focus on identifying year of study factors so as better to understand how year of study interacts with persistent neck pain.

A higher percent time of computer use for entertainment reduced the risk of persistent neck symptoms. Computer use for entertainment included activities such as chatting, playing games, listening to music, and watching movies. Hakala et al [54] suggested that the basic mechanism of computer game playing mostly required repetitive hand motion in sitting position and dynamic action where players change postures freely, thereby minimizing loading of the upper extremities. Thus computer use for entertainment may not require the user to be in static postures for prolonged periods. It is also plausible that computer use for entertainment reduces mental stress [55]. Bongers et al [56] suggested that psychosocial demands can exceed an individual's coping capabilities, resulting in a stress response that in turn could produce muscle tension, static loading of muscles, or generate other physiological responses that may result in musculoskeletal symptoms.

A keyboard position self-rated as too high increased the risk of persistent neck pain. Faucett et al [57] found that higher keyboard height (with respect to elbow height) was associated with increased risk of neck, upper back, and upper extremity discomfort. Mekhora et al [58] found that neck and shoulder discomfort significantly declined when keyboard level was adjusted to suit the individual's comfort and proposed that adjustment of keyboard level would reduce the need to reach the hand forward and backward or to elevate the shoulder. Therefore, less muscle activity would be present after the adjustment.

Interestingly, no psychosocial and clinical domains remained in the final model. Although it is currently accepted that a large index number within psychosocial dimensions contributes to onset and persistence of musculoskeletal symptoms in adults [59], evidence for such effect in the young population is far from conclusive. Grimby-Ekman et al [14] found that perceived stress was a risk factor for developing neck or upper back pain and for persistent neck or upper back pain in Swedish undergraduate students. On the other hand, Hanvold et al [8] reported no association between stress level and incidence of neck pain during 3-year follow-up among technical school students. In the present study we only examined a selected group of psychosocial and clinical factors. Other important psychosocial and clinical factors may be identified in future work.

The major strength of this study is its prospective design and the evaluation of broad biopsychosocial factors for their contribution to neck pain. However, the study has several weak points. First, the nature of several biopsychosocial factors and the diagnosis of neck pain were subjective, which may have led to inaccuracy. Future studies should consider inclusion of objective information to increase accuracy. Second, information regarding frequency and severity of pain was not collected in this study. Further study should gather this information to enhance understanding regarding relations between risk factors and musculoskeletal symptoms in undergraduate students. Third, subjects in this study were recruited only from one university. Thus generalization of the results to other undergraduate student populations may be limited.

## Conclusions

Among a large sample of undergraduate students 46% reported neck pain during a 1-year period of follow-up, of whom 33% experienced persistent neck pain. Very few proposed risk factors were found to predict onset and persistence of neck pain, which included certain aspects related to computer use and year of study. One strategy to prevent morbidity in adults should focus on the health of undergraduate students. An education program should be introduced for undergraduate students regarding how properly to do computer work to avoid neck pain.

## Competing interests

The authors declare that they have no competing interests.

## Authors' contributions

All authors had access to the data. SK provided concept/research design, data collection, data analysis and manuscript writing. PJ provided concept/research design, data analysis and manuscript writing. PP and WJ provided concept/research design and manuscript writing. All authors read and approved the final manuscript.

## Pre-publication history

The pre-publication history for this paper can be accessed here:

http://www.biomedcentral.com/1471-2458/11/566/prepub
